# Simulation of Contraceptive Access for Adolescents and Young Adults Using a Pharmacist-Staffed e-Platform: Development, Usability, and Pilot Testing Study

**DOI:** 10.2196/60315

**Published:** 2025-02-19

**Authors:** Kayla Knowles, Susan Lee, Sophia Yapalater, Maria Taylor, Aletha Y Akers, Sarah Wood, Nadia Dowshen

**Affiliations:** 1 PolicyLab Children's Hospital of Philadelphia Philadelphia, PA United States; 2 Clinical Futures Children's Hospital of Philadelphia Philadelphia, PA United States; 3 Craig Dalsimer Division of Adolescent Medicine Children's Hospital of Philadelphia Philadelphia, PA United States; 4 Division of General Internal Medicine, Department of Medicine University of Pennsylvania Philadelphia, PA United States; 5 Stellar Pharmacy Services Inc Avondale, PA United States; 6 Guttmacher Institute New York, NY United States; 7 Department of Pediatrics, Perelman School of Medicine University of Pennsylvania Philadelphia, PA United States

**Keywords:** adolescent, contraception, telemedicine, user-centered design, young adult, reproductive, design, usability, experience, mHealth, mobile health, app, youth, teenager, drug, pharmacology, pharmacotherapy, pharmaceutics, medication, pharmacy, digital health, platform, access

## Abstract

**Background:**

Offering contraceptive methods at pharmacies without a prescription is an innovative solution to reduce the incidence of unintended pregnancies among adolescents and young adults (AYA). Pharmacy-prescribed contraception may increase the convenience, simplicity, and affordability of contraceptives.

**Objective:**

The aim of this study was to develop, pilot test, and evaluate the acceptability and feasibility of a telemedicine electronic platform app simulating pharmacist prescribing of contraceptives to AYA as well as assess agreement between pharmacist-simulated contraceptive approvals and contraception as prescribed in routine clinic visits.

**Methods:**

This study was conducted in two phases: (1) development and usability testing of a prototype app to simulate pharmacists prescribing contraceptives to AYA and (2) pilot testing the app in a simulation for AYA requesting contraception from a pharmacist with pharmacist review and request approval or rejection. Eligibility criteria in both phases included the following: assigned female sex at birth, age 15-21 years, seeking contraceptive services at an academic adolescent medicine clinic, prior history of or intention to have penile-vaginal intercourse in the next 12 months, smartphone ownership, and English language proficiency. Phase 1 (usability) involved a video-recorded “think aloud” interview to share feedback and technical issues while using the app prototype on a smartphone and the completion of sociodemographic, sexual history, and perception of the prototype surveys to further develop the app. Phase 2 (pilot) participants completed phase 1 surveys, tested the updated app in a simulation, and shared their experiences in an audio-recorded interview. Descriptive analyses were conducted for quantitative survey data, and thematic analyses were used for interview transcripts.

**Results:**

Of the 22 participants, 10 completed usability testing, with a mean age of 16.9 (SD 1.97) years, and 12 completed pilot testing, with a mean age of 18.25 (SD 1.48) years. Three issues with the prototype were identified during “think aloud” interviews: challenges in comprehension of medical language, prototype glitches, and graphic design suggestions for engagement. Usability testing guided the frontend and backend creation of the platform. Overall, participants agreed or strongly agreed that using an app to receive contraceptives would make it easier for teens to access (n=19, 86%) and make contraceptive use less stigmatizing (n=19, 86%). In addition, participants agreed that receiving contraception prescriptions from a pharmacist without a clinic visit would be safe (n=18, 82%), convenient (n=19, 86%), acceptable (n=18, 82%), and easy (n=18, 82%). Pharmacists and medical providers had 100% agreement on the prescribed contraceptive method for pilot participants.

**Conclusions:**

AYA found contraceptive prescription by a pharmacist via an app to be highly acceptable and provided critical feedback to improve the design and delivery of the app. Additionally, pharmacist contraceptive approvals and contraception as prescribed in routine clinic visits were identical.

## Introduction

Pregnancy rates among adolescents and young adults (AYA) 15-19 years old have dropped from 61.8 births per 1000 in 1991 to 13.5 in 2022, while AYA 20-24 years old reported a record low pregnancy rate of 60.4 births per 1000 [1]. For 2010-2019, pregnancy rates declined the most for youth 19 years old and younger, a 50% decrease, followed by a 29% drop for 20-24 year olds [2]. Evidence suggests the decline in pregnancy rates may be attributed to increased access to comprehensive sex education, use of contraceptives and health care, and not due to decreased sexual activity [3]. However, sexually active AYA, aged 15-24 years, were the most likely age group to experience unintended pregnancies in the United States [2,4]. Despite evidence showing the value of access to sex education and contraceptive services in reducing pregnancy rates among AYA, laws restricting access to both are increasingly being introduced and passed in US state legislatures. Legal restrictions intensify barriers, such as cost, attending appointments, stigma, and more to accessing contraceptives [5,6], which disproportionately affects low-income, disabled, racial or ethnic minorities, and other marginalized women and other birthing people [7]. Pharmacist-prescribed contraception—a strategy already used in high-income and many low- and middle-income countries but rarely in the United States—is one such strategy. In 2019, the American College of Obstetricians and Gynecologists recommended pharmacist-prescribed contraception without age restrictions as a necessary step to increase over-the-counter access to hormonal contraception and reduce the rate of inconsistent or nonuse of contraception [8].

In prior research, women and other birthing people voiced “convenience, simplicity, [and] affordability” as primary benefits of pharmacist-prescribed contraception [9]. In a study in California among 426 women and other birthing people, pharmacy access for emergency hormonal contraceptive (EHC) was preferred to clinician prescription as it was perceived to be faster (54%) and more convenient (47%) than seeking physician prescription [10]. Another study found that 68% of women who were at risk for unintended pregnancy reported they would prefer to obtain the contraceptive pill, patch, ring, or EHC from a pharmacy rather than a clinic if pharmacist prescribing was an option [9]. Moreover, 41% of those not currently using contraception reported they would start a contraceptive method via pharmacist prescribing if available [10]. Provision of EHC via community pharmacies has increased the use of EHCs; moreover, expanding access in this way is estimated to prevent almost half (1.3 million) of the 3 million unintended pregnancies annually [11]. Centering patient priorities for access, as well as patient preferences for method choice, is a key tenet of reproductive justice and high-quality contraceptive care.

Historically, women and other birthing peoples’ safety has been the most common concern regarding contraception delivery without a clinician-provided prescription [9]. However, multiple studies demonstrate that patients can accurately self-screen for contraindications to contraceptive use using medical checklists [12,13]. One study found greater than 93% of 328 patient-physician concordance for risk factor identification [12]. In another study, self-screening by patients using a medical checklist of contraindications was found to have greater sensitivity (83.2%) and specificity (88.8%) than a patient self-completed clinician questionnaire, which asked them to simply consider their medical history to determine the presence of contraindications (56.2% sensitivity; 57.6% specificity) [13]. This means when using a medical checklist of contraindications, women were able to accurately self-screen for contraindications to combined hormonal contraception [13]. Given the consistency of evidence supporting patient ability to medical self-screen for contraceptive contraindications, the American College of Obstetricians and Gynecologists endorses patient use of self-screening tools to determine eligibility for over-the-counter access to hormonal contraception [8].

Pharmacist-prescribed contraception innovations will need to develop strategies for successful implementation prior to widespread scaling [14]. Implementing screening tools has proved challenging for non-sexual health services for select populations, like youth [14]. Developing outreach strategies for youth and other vulnerable populations may require careful consideration. Attention to the training of point-of-service staff may facilitate service delivery and uptake. Studies of pharmacy staff indicate greater hesitancy and a desire for more intense training before providing sexual health services compared to non-sexual health services [15]. Attention to training and post-training support services may be necessary to ease implementation challenges. Newer technologies, like telemedicine, that allow skilled providers to deliver services across a distance may be valuable in bridging this gap until larger numbers of pharmacists are comfortable delivering pharmacist-prescribed sexual health services. Combining innovations such as telehealth and pyxis machines can allow pharmacists who are trained and comfortable providing sexual health services to AYA to do so.

There is limited research on pharmacist-prescribed contraception for US adolescents [4]. This study sought to develop an e-platform app called Birth Control Pass (BCPass) to simulate pharmacists prescribing contraceptives to AYA, test the acceptability and feasibility of pharmacist-delivered contraception among AYA as a proof of concept, and determine the concordance between pharmacists and providers on the appropriate contraceptive method(s) to be prescribed to participants.

## Methods

### Overview

This study occurred in 2 phases. In Phase I, the e-platform was developed, and participants were recruited to engage in usability testing. In Phase 2, participants pilot-tested the e-platform.

### Participants and Setting

Eligible participants for both study phases were patients seeking contraceptive initiation services at a subspecialty academic adolescent medicine clinic, ages 15-21 years old, assigned female sex at birth, with a prior history of or intention to have penile-vaginal intercourse in the next 12 months, owned a smartphone and could read and speak English. Usability testing of the prototype was completed in April of 2021. Modifications were made to the app to address participants’ concerns and implement suggestions through iterative usability testing between developers and the study team. Pilot testing of the final prototype occurred between October 2021 and August 2022.

### Ethical Considerations

Institutional Review Board approval was granted by the Children’s Hospital of Philadelphia (20-017957). Participants received a $20 US gift card for their time and effort. Precautions were taken to secure participants' personal information to ensure confidentiality including, the use of study identification numbers that were assigned to participants and used in place of participants' name and other private information on data collection forms.

### Recruitment Strategy

For both study phases, the study team reviewed clinic schedules daily to identify patients with contraceptive appointments on the same day or the following clinic day. Clinic staff also referred patients for recruitment. Patients were approached in person at their medical appointment, by phone call, or via SMS text message. Consent was obtained via wet-ink signature on paper forms or electronic consent (e-consent) on Children’s Hospital of Philadelphia’s Research Electronic Data Capture (REDCap) [16,17] data collection application. Consent was provided either by legal guardians who attended clinic visits with patients 17 years old and younger or by the patients themselves, who were legally eligible to consent for themselves if they attended clinic visits alone [18] or were older than 18 years old. Participants were informed that by engaging in the research study, they would be testing the e-platform interface that simulated pharmacist prescribing but that they would not receive contraception as part of the study activities. Participants understood that contraception would be provided by their clinician during their scheduled medical visit, as per usual clinical care guidelines. Participants engaged with the e-platform either before or after their scheduled medical visit.

### Study Procedures

#### Phase 1: Development and Usability Testing

We created the BCPass prototype by modifying a large pediatric hospital system’s app for employee COVID symptom check-in. The goal of the prototype was to simulate patient medical screening, patient contraceptive choice, and pharmacist contraceptive prescribing. The prototype first collected contact and self-screening information related to background and medical history. Next, participants were instructed to review content about contraceptive methods using a direct link to the educational website bedsider.org [19] and indicate their preferred method. Responses on the self-screening form were linked directly to medical contraindications to contraceptive prescribing per the Centers for Disease Control and Prevention US Medical Eligibility Criteria to facilitate rapid prescribing decision-making [20]. The prototype app included only a front-end interface for usability testing participants, but there was no backend personal health information data storage or pharmacist involvement. The study team explained to participants how the final app would work, including that if the participant had questions during the simulation, they had the option to call a pharmacist via the app (“Ask a Pharmacist” call button). Additionally, once the request for the preferred contraceptive method was submitted, a pharmacist would review and complete a “Pharmacist Approval Assessment Form” indicating if they would prescribe the contraceptive and approve dispensing or reject the request.

**Figure 1:**
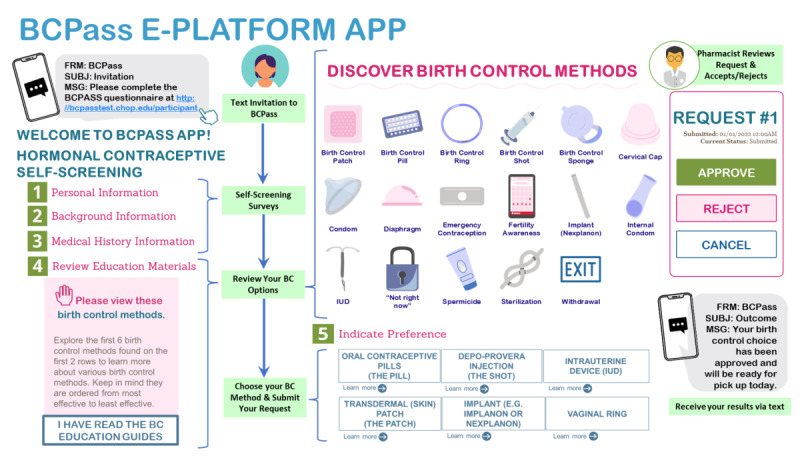
Birth Control Pass (BCPass) e-platform app overview. Overview of the BCPass app, invite link, participant-entered information, education information review [19], preferred contraception option, and the pharmacist review and outcome. "Discover Birth Control Methods" was adapted from the bedsider.org birth control dashboard webpage [19].

Participants completed web-based multiple-choice and short-answer surveys regarding their sociodemographic, sexual history, and attitudes regarding pharmacist-prescribed and app-delivered contraception. Next, they tested the prototype in a video-recorded “think aloud” interview [21], capturing the participants’ initial impressions of the prototype, technical issues, and feedback while they actively engaged with the app prototype on their own smartphones*.* Studies using the “think aloud” methodology have proven to be successful at identifying usability problems without requiring a large number of subjects [21]. Participants were prompted to remark on ease of use, design aspects that are confusing or that slow task completion, and graphical elements, such as font size, ratios of images to words, and color schemes. The study team observed this process and took structured notes to capture information regarding domains from the sociotechnical model to ensure the optimization of service delivery [22]. Following prototype testing, participants’ attitudes toward receiving contraception from a pharmacist and an app were assessed on a 5-point Likert scale: 1=strongly disagree, 2=disagree, 3=unsure, 4=agree, and 5=strongly agree. A 7-point Likert scale was used to evaluate the usefulness, ease of use, effectiveness, reliability, and satisfaction with the prototype (1=strongly disagree, 2=disagree, 3=somewhat disagree, 4=neither agree nor disagree, 5=somewhat agree, 6=agree, 7=strongly agree). Analyzed usability testing data was presented to the study team and app developers to inform the development of the app frontend and backend, along with iterative testing and weekly meetings (Figure 1). Special care was taken to thoroughly test each “click” on the app, and automated messages were reviewed to ensure ease of access and fluidity.

#### Phase 2: Pilot Testing

Once a fully functional app was developed, a study pharmacist was trained to implement the study protocol, and AYAs were recruited to pilot test the intervention. Pilot participants completed web-based surveys regarding their sociodemographic, sexual history, and attitudes toward receiving contraception from an app and a pharmacist. Next, participants completed a simulation exercise, which included completing a medical history form to identify contraindications to contraception, learning about contraceptive options via bedside.org [19], and selecting a contraceptive method. As part of the simulation, a text or email was sent to their smartphone with a link to the app. After they submitted their request for a contraceptive method, the study pharmacist accessed the request on the backend to review and complete the “Pharmacist Approval Assessment Form.” Participants then received an automated SMS text message to confirm if the pharmacist accepted (prescribed the contraceptive and approved dispensing), rejected (shared information to call the clinic that provides contraceptive care), or requested additional information (when participants requested methods unavailable for pharmacist prescribing, such as long-acting reversible contraceptives and injectable medroxyprogesterone acetate).

Following the simulation, pilot participants completed a web-based survey to evaluate the usefulness, ease of use, effectiveness, reliability, and satisfaction on 5-point and 7-point Likert scales. Participants then engaged in a brief audio-recorded interview that solicited feedback on the app and participants’ decision-making process in selecting a contraceptive method. After participants completed their clinic visit, the contraceptive method prescribed during their routine appointment was abstracted from their electronic medical record.

#### Analysis

Data analysis was conducted at two time points following phase 1 and phase 2, respectively. Deidentified survey demographics, sexual history, and Likert responses were exported from REDCap to Microsoft Excel for analysis. Descriptive analysis was computed for demographics and sexual history (proportions), and mean score and standard deviation were calculated for Likert questions. Audio from the “think aloud” video interview (usability) and feedback interview (pilot) were transcribed and manually coded by two coders until a 95% agreement regarding the themes was reached. For usability testing, participant comments and suggestions were categorized based on the type of modifications needed to the app (ie, wireframe, self-screening comprehension, and graphics). Interview data from the pilot testing was similarly thematically coded to reflect perceptions about the app’s effectiveness, acceptability, feasibility, and participants’ contraceptive decision-making. Effectiveness was defined as the e-platform success in simulating a birth control prescription. Acceptability or satisfaction with the e-platform was defined as participants’ perceptions when using BCPass. Feasibility (BCPass practicality for learning about and accessing birth control) was defined by BCPass’s usability or ease of use in navigating the e-platform and submitting a request for birth control. Lastly, birth control decision-making was based on participants’ considerations made when indicating their birth control preference before and after using BCPass. Using the codebook, two coders not involved in usability qualitative analysis (one assisted with e-platform modifications and data collection, and the other helped with qualitative analysis only) classified statements in each thematic area as positive, neutral, or negative. Finally, we assessed concordance between the contraceptive method selected at the medical visit (electronic medical record data) and the pharmacists’ decision to approve or reject the participant’s request for a contraceptive method.

## Results

### Participant Characteristics

In total, 22 AYA participated: 10 in phase 1 (usability testing) and 12 in phase 2 (pilot testing). On average, participants were 17.64 (1.50) years old, had some high school education (n=13), graduated from high school (n=4) or had some college (n=5), and had previously used contraception (n=17). See Table 1 for participant demographics and sexual history.

**Table 1:** Usability and pilot testing participant demographic and sexual history.

Characteristics		Usability testing (n=10)	Pilot testing (n=12)	Totals (N=22)
**Age (years), n (%)**				
	15-16	3 (30)	1 (8)	4 (18)
	17-18	7 (70)	7 (58)	14 (64)
	19-21	0 (0)	4 (34)	4 (18)
**Race or ethnicity, n (%)**				
	Black or African American	Unknown^a^	6 (50)	Unknown^a^
	Hispanic or Latinx	Unknown^a^	1 (8)	Unknown^a^
	White (non-Hispanic or Latinx)	Unknown^a^	5 (42)	Unknown^a^
**Education, n (%)**				
	Not graduated high school	8 (80)	5 (42)	13 (59)
	High school degree or General Education Development	1 (10)	3 (25)	4 (18)
	Some college	1 (10)	4 (33)	5 (23)
**Number of sex partners, n (%)**				
	None	4 (40)	2 (17)	6 (27)
	Less than 3	3 (30)	8 (66)	11 (50)
	Between 3 and 5	2 (20)	0 (0)	2 (9)
	More than 5	0 (0)	2 (17)	2 (9)
	Preferred not to say	1 (10)	0 (0)	1 (5)
**Ever taken a pregnancy test, n (%)**				
	No	6 (60)	6 (50)	12 (55)
	Yes	4 (40)	6 (50)	10 (45)
**Ever been pregnant, n (%)**				
	No	10 (100)	12 (100)	22 (100)
	Yes	0 (0)	0 (0)	0 (0)
**Used birth control to prevent pregnancy, n (%)**				
	No	3 (30)	2 (17)	5 (23)
	Yes	7 (70)	10 (83)	17 (77)
**Ever used condoms during sex, n (%)**				
	No	3 (30)	2 (17)	5 (23)
	Yes	7 (70)	10 (83)	17 (77)
**Frequency of condom use^b,c^, n (%)**				
	Never	0 (0)	1 (10)	1 (6)
	Sometimes	2 (29)	6 (60)	8 (47)
	Always	4 (57)	3 (30)	7 (41)
	Unknown	1 (14)	0 (0)	1 (6)
**Ever had a sexually transmitted infection^b^, n (%)**				
	No	8 (80)	8 (67)	16 (73)
	Yes	2 (20)	3 (25)	5 (23)
	Unknown	0 (0)	1 (8)	1 (4)

^a^Race or ethnicity was not self-reported by usability participants in this phase of the study. Electronic medical record race and ethnicity data is not self-reported and may misidentify participants. Therefore, this study only reports self-reported information collected during the pilot testing phase.

^b^Due to rounding, some totals may not correspond with the sum of the separate figures.

^c^Participants were only asked about their frequency of condom use if they answered yes to using condoms during sex. Participants could have responded yes to ever using condoms, but they may not currently be using them.

### Quantitative Survey: Acceptability and Feasibility of Pharmacist- and App-Delivered Contraception

#### Pharmacist-Prescribed Contraceptives

Usability and pilot participants (N=22) agreed receiving a contraceptive prescription from a pharmacist without a clinic visit would be safe (mean 4.27, SD 0.88), convenient (mean 4.50, SD 0.74), easy (mean 4.32, SD 0.89), and acceptable (mean 4.27, SD 1.08; Table 2). If they had the option to receive contraception from a pharmacist without a clinic visit, 14 of 22 participants reported they were likely or very likely to do so.

**Table 2:** Perceptions of pharmacist and app-delivered contraception (N=22, Likert Scale 1-5).

		Mean (SD)
**Safety, convenience, acceptability, and ease receiving birth control**		
	Getting birth control prescribed by a pharmacist would be safe	4.27 (0.88)
	Getting birth control from a pharmacist would be convenient	4.50 (0.74)
	Getting birth control from a pharmacist would be acceptable to me	4.27 (1.08)
	I think it would be easy to receive birth control from a pharmacist	4.32 (0.89)
	Getting birth control from an app would be safe	3.82 (1.14)
	Getting birth control from an app would be convenient	4.45 (1.01)
	Getting birth control from an app would be acceptable to me	3.95 (1.17)
	I think it would be easy to use an app to get birth control	4.36 (1.00)
**Advantages of using an app to receive birth control**		
	It would be easier for teenagers to get oral contraceptives	4.55 (0.74)
	It would feel less embarrassing	4.27 (0.98)
	It is less stigmatizing, meaning more normal to use	4.45 (0.86)
	Fewer teenagers would get pregnant	4.32 (0.99)
	It would be more confidential	3.82 (1.30)
**Disadvantages of using an app to receive birth control**		
	Teenagers might not use condoms to protect against sexually transmitted diseases	3.91 (0.87)
	Teenagers need a doctor to decide if oral contraceptives are safe for them	3.73 (1.12)
	Teenagers might have sex at a younger age	3.14 (1.32)
	Teenagers might use oral contraceptives incorrectly	3.36 (1.05)
	Teenagers might not get tested for sexually transmitted diseases	3.55 (1.26)
	Oral contraceptives might cost more over the counter	3.50 (0.86)
	Teenagers might not talk to their parents about birth control	4.00 (0.87)
	I have no worries (concerns) about teens using a medication dispensing machine to get birth control	3.09 (1.11)
**Social approval**		
	Most people who are important to me would approve of me using an app to get birth control	3.64 (1.14)
	Most teens like me would use an app to get birth control	4.27 (0.77)
	Teens my age would use an app to get birth control	4.23 (0.97)
	Parents or family would support me using an app to get birth control	3.32 (1.30)
	My romantic partner(s) would support me using an app to get birth control	4.09 (1.06)
	The decision to use an app to get birth control would be totally up to me	4.18 (1.10)
	I am confident that I could use an app to get birth control	4.09 (1.19)

#### Receive Contraceptives Through an e-Platform App

Usability and pilot participants (N=22) agreed that receiving contraception using an app would be safe (mean 3.82, SD 1.14), convenient (mean 4.45, SD 1.01), easy (mean 4.36, SD 1.00), and acceptable (mean 3.95, SD 1.17) (Table 2). Additionally, participants were confident they would be able to use an app to get contraception (mean 4.09, SD 1.19) and agreed most teens would use an app to get contraception (mean 4.27, SD 0.77). A potential advantage recognized by participants of using an app would include fewer teenagers experiencing an unintended pregnancy (mean 4.32, SD 0.99), getting contraception would be less embarrassing (mean 4.27, SD 0.98), and less stigmatizing (mean 4.45, SD 0.86). Participants agreed a potential disadvantage of receiving birth control from an app is that it may lead teens to not talk to their parents about birth control (mean 4.00, SD 0.87) or a possible decrease in condom use and an increase in sexually transmitted infections among teens (mean 3.91, SD 0.87). See Table 2 for additional insight into participants’ attitudes toward pharmacists and app-delivered contraceptives.

#### E-Platform App Survey Feedback

Overall feedback on the BCPass app simulation during prototype and final productive testing was positive (Table 3). Participants agreed that BCPass was simple to use (mean 6.33, SD 1.24) and pleasant to interact with (mean 6.00, SD 1.64). Additionally, participants felt the app could do everything they would want it to be able to do for contraceptive delivery (mean 6.15, SD 1.23) and agreed they would use it again (mean 6.29, SD 1.54).

**Table 3:** BCPass app simulation feedback (N=22, Likert scale 1-7).

	Mean (SD)
BCPass was simple to use	6.33 (1.24)
BCPass was easy to learn and use	6.43 (1.02)
I believe I could become productive quickly using BCPass	6.05 (1.51)
The way I interact with BCPass is pleasant	6.00 (1.64)
I like using BCPass	6.00 (1.44)
BCPass can do everything I would want it to be able to do	6.15 (1.23)
I would use BCPass again	6.29 (1.54)

#### Clinician- and Pharmacist-Prescribed Contraception Concordance

A comparison of piloting participants who indicated a contraceptive preference (n=9) and what was prescribed revealed pharmacist contraceptive decisions and contraception methods as prescribed in routine clinic visits were identical. One of the 8 participant requests was rejected by the pharmacist because of a contraindication. This participant noted in their feedback interview that they requested a method they knew was contraindicated because it was their first choice over the contraception method they were prescribed. Three participants selected “I don’t know” at the end of the simulation. One remained undecided following their clinic appointment. Another selected “I don’t know” because they had an IUD at the time of the study but knew their first-choice method was contraindicated. The third selected a method at their clinic appointment. Of the 12 participants, one did not attend their clinic appointment, so it is unknown what they would be prescribed.

### Qualitative Feedback

#### Usability Testing Think Aloud Data and Resultant e-Platform App Modifications

In an analysis of usability testing phase data, participants identified modifications related to addressing prototype wireframe glitches, self-screening comprehension, educational resource engagement, and app aesthetics. The prototype’s wireframe glitches were anticipated as it was designed as a temporary test environment, and once the production frontend, patient-facing screen, backend, and developer view were created, the issues were resolved.

While navigating the prototype, participants requested clarification on the medical history questionnaire, indicating comprehension concerns. For example, participants asked if the transdermal skin patch was the same patch with which they were familiar. Additionally, participants asked what constitutes prolonged immobility or a bad reaction to hormonal contraception. The study team revised any questions that were identified as confusing and may be perceived as confusing to participants’ peers. Examples and lay language were used for questions that required more medical terminology, such as including the more popular names for contraceptive methods in parentheses next to the full names (ie, oral contraceptive pill [“the pill”], transdermal hormonal patch [“the patch”], and injectable medroxyprogesterone acetate [“the shot”]).

Participants’ feedback on the lack of educational material and the app’s aesthetics were closely associated concerns. Numerous participants noted they skipped the mandatory contraceptive method educational website, explaining they did not see the link or felt it was not important. The color palette and ambiguity of the “Ask a Pharmacist” call button and bedsider.org weblink were noted as weaknesses. The team determined adding color and images was a solution to attract participants’ attention to educational materials. A welcome page was created to introduce the purpose of BCPass and provide written instructions on how to contact the study pharmacist via the “Ask a Pharmacist” call button.

#### Pilot Testing Interview Feedback

The following four primary themes emerged during the coding process: (1) the perceived effectiveness of the BCPass app, (2) AYA’s perceptions of using BCPass (usefulness as a standalone or compared to the standard of care), (3) BCPass practicality for learning about and accessing contraception via an app, and (4) AYA’s considerations when selecting a contraceptive method before and after the BCPass simulation.

Of the 12 pilot participants, one interview was not completed because the study team could not reach the participant; thus, the results reflect 11 of the 12 participants. A quarter of the 11 pilot participants commented on receiving contraceptive prescriptions through BCPass from a pharmacist easily in the simulation. One participant expressed that BCPass may be unrealistic outside of the simulation due to safety concerns they had in making contraception more accessible to AYA without additional educational discussions with a provider. Due to the nature of the simulation, participants understood they would not receive a method as part of the study; therefore, the perceived effectiveness of BCPass was minimally mentioned.

“The only thing that surprised me was that um you can literally just just get it. Like you don’t like need any doctor’s approval like just being able to get it”

Participant 1, selected implant in BCPass

BCPass was highly acceptable among the 11 AYA pilot participants. The majority of pilot participants (73%) found BCPass to be quick, easy, convenient, and accessible. More than half (64%) found the process to be enjoyable and a good accessible option for other AYAs to request contraception compared to the standard of care of receiving contraception from a clinician after a medical appointment. Many participants stated they liked receiving contraception information from BCPass as well as avoiding a trip to a clinic and potentially uncomfortable conversation with a clinician (55%). Yet 18% of participants expressed they would also like the option to talk to a clinician by phone or in person in addition to the option they were given to talk to a pharmacist through BCPass. Some participants (18%) felt BCPass may not be adequate for AYA due to the possible need for more direct clinician oversight for young people and those with complex care needs.

“I also really liked the idea of not having to go to the doctor and have an awkward conversation about getting birth control”

Participant 2, selected oral contraceptives in BCPass

“I like that it was fast and convenient and I didn’t have to go see a doctor. I could do it right on my phone. Um, I liked that it like also gave me a little feel about all the different kinds of birth control methods because I think that we are not often given information about all of them”

Participant 3, selected IUD in BCPass

A majority of the 11 pilot participants (91%) reported that BCPass was easy, quick, and convenient to use, with straightforward instructions and easy-to-answer questions. A third (36%) stated the information they received as part of the BCPass simulation was comparable to what they received at a medical appointment, including a participant stating the BCPass questions and education materials helped them determine what contraceptive method was best for them. While many usability concerns were addressed in the first phase of the study, 64% of participants shared feedback to further improve the experience for AYA. Suggestions included shortening the questionnaire, clarifying questions, deleting repetitive questions, and improving the delivery of educational materials.

“I liked how, I enjoyed how easy and self-explanatory a lot of the questions get… Sorry about that. I enjoyed mostly the descriptive questions that I was asked so that I was more sure about the pathway I would like to take while using that”

Participant 4, selected Depo-Provera injection in BCPass

Finally, participants were asked about their preferred contraceptive method selected using BCPass. Most of the 11 (82%) pilot participants reported prior use of the method they chose in the BCPass simulation. More than half (64%) cited their medical history and side effects associated with their preferred method when explaining their decision. Additional considerations participants noted were the delivery of contraception (eg, shot, long-acting reversible contraceptives, daily oral pill) (27%) as well as discussions they have had with family and friends (27%).

“I felt like it [the patch] was the easiest one to remember and it was something I didn’t have to take every day and was something I only have to remember once a week. I feel like it would be easy on my time and my hobbies I tend to forget about stuff”

Participant 5, selected transdermal patch in BCPass

Prior to the BCPass simulation, two of the 11 (18%) pilot participants stated they did not have a single preferred contraceptive method in mind. After completing the simulation, they were able to more confidently select the method that they felt would work best for them. These participants credited the BCPass simulation questionnaire and educational materials in their decision-making process.

“Um I would definitely say that like IUD now. Originally, I would say 50-50 percent, like I would’ve leaned either way. But now using the app I would definitely say that I would have rather used the IUD because it’s more, I don’t want to say more protective. But like, it’s better, it’s like safer, better protection”

Participant 3, selected IUD in BCPass

## Discussion

### Principal Findings

We found that an app for pharmacist delivery of contraception, BCPass, was acceptable and usable for AYA. Participants liked how easy, convenient, and fast BCPass was to request contraception. Once the concerns addressed in the usability phase were incorporated, there were no glitches experienced, and few participants felt the app survey was too long or had confusing questions. Also, more education may be needed to ensure AYAs feel safe requesting contraception from a pharmacist. Finally, we identified 100% agreement between pharmacist contraceptive approvals in the simulation and contraception as prescribed in routine clinic visits.

Our findings are consistent with prior research on this topic in older adult women. In one survey study, women and other birthing people (≥18 years) reported receiving contraception from a pharmacist may be more convenient, faster, and easier compared to getting contraception from a clinician [10], which mirrors AYA’s beliefs in this study. Additionally, this study found AYA-reported advantages of using an app included improving access to contraception by reducing the stigma and embarrassment of using contraception, reducing the burden of scheduling and attending clinic visits, and reducing the number of teenagers who become pregnant. In our study’s feedback interview, most pilot participants emphasized their favorite aspect of BCPass was how quick and convenient it was compared to the standard of care while still receiving the contraceptive information they desired.

We did not identify any discordance between simulated pharmacist approvals and clinicians, as prescribed in clinic visits, suggesting the safety of having youth self-screen for medical contraindications. Women and other birthing people have previously been shown to safely and accurately complete self-screening medical history questionnaires and identify contraindications [12,13]. Like the older cohort, this study has shown AYA were capable of reporting their medical history, reviewing education materials, and selecting a contraceptive method they wish to use without forgoing a medical professional’s review. While this study made edits, such as including more lay language and providing examples to some questions, to the medical contraindications to contraceptive prescribing per the Centers for Disease Control and Prevention US Medical Eligibility Criteria [20], once these changes were made to accommodate AYA’s readability and comprehension level, they were able to successfully communicate their own medical history with a pharmacist through the app. A few pilot participants noted in their feedback interview they were surprised how easy completing the simulation was because questions regarding medical history were primarily all yes or no with an option to write in additional information for context with the study pharmacist. Some participants even found questions on their contraception preferences and reviewing educational materials more helpful in picking their preferred contraception than speaking to a clinician. Additionally, the feedback from the pilot phase reflected AYA’s thoughtful consideration and awareness of their medical history, as well as side effects when planning to request contraception.

Of note, only one participant expressed concerns that contraception prescription without a clinician’s oversight could be dangerous for AYA by making contraception too accessible, but several felt that they would prefer to discuss it with a provider. This data suggests the BCPass educational materials and access to call a pharmacist were not as effective as they needed to be to assure safety. While most participants liked the educational materials, a few did not feel it was enough to educate AYA on side effects. This finding suggests a need to include multimodal educational material, including more visual and audio materials. Additionally, participants who questioned the safety of BCPass also did not view pharmacists as being as knowledgeable as clinicians in prescribing contraception, which contrasts with physicians and midlevel practitioners in other studies who have supported pharmacist-initiated access to contraceptives [15]. Safety concerns indicated educational information presented needs to not only address contraception concerns but also inform AYA of the pharmacist’s credibility and remind future users that pharmacists provide medical care already, such as vaccine delivery and drug interaction information [23].

Limitations of this study include the small sample size, single-city site, and limited rounds of usability testing. In addition, current state policy allows only for the simulation of pharmacist prescriptions. Following usability testing and additional app modifications, the platform was not retested again in another usability round before it was launched in the pilot testing phase. We focused on contraceptive methods for pregnancy prevention only and did not include the myriad of other reasons AYA may use contraception (eg, menstruation management, acne treatment, gender-affirming care, and medical conditions such as endometriosis). In addition, some participants completed the BCPass simulation following their medical appointment, which was not documented but is an important consideration for future larger-scale testing of the simulation. Future studies should further inform apps’ usefulness in improving access to contraception for a broad range of indications via pharmacist prescription. Additionally, future research should explore the acceptability and feasibility of BCPass for key populations, including but not limited to girls, women, and other birthing people who are low income, living with disabilities, and sexual and gender minorities, to navigate barriers to accessing contraception. At the time of this study, pharmacist dispensing of contraceptives was not legal in the state of Pennsylvania, where this study took place, and 29 other US states. During a time with increasingly restrictive laws on connecting AYAs to contraceptives and other family planning resources, implementing methods to increase access to contraception is more important than ever because AYAs are particularly vulnerable to these restrictions.

### Conclusions

Our results indicate that the BCPass app has the potential to be a valuable tool to improve access to contraception more equitably by facilitating contraceptive education, person-centered decision-making, and convenient delivery. BCPass and other electronic health solutions can supplement traditional care models and can be easily scalable, time-efficient, and cost-effective to assist AYA with navigating barriers to accessing contraceptives. Our results, demonstrating high acceptability and usability, suggest the potential of apps as supplemental effective tools to expand access to contraception for AYA during this time of increasing restrictive laws and policies impacting AYA reproductive health.

## Abbreviations

AYA: adolescents and young adults
BCPass: Birth Control Pass
EHC: emergency hormonal contraceptive
REDCap: Research Electronic Data Capture

## References

[1] Hamilton BE, Martin JA, Osterman MJK (2023). Births: Provisional Data for 2022, in Vital Statistics Rapid Release. Centers for Disease Control and Prevention.

[2] Rossen LM, Hamilton BE, Abma JC (2023). Updated Methodology to Estimate Overall and Unintended Pregnancy Rates in the United States. Centers for Disease Control and Prevention.

[3] Lindberg L, Santelli J, Desai S (2016). Understanding the decline in adolescent fertility in the United States, 2007-2012. J Adolesc Health.

[4] Finer LB (2010). Unintended pregnancy among U.S. adolescents: accounting for sexual activity. J Adolesc Health.

[5] Swan LET (2021). The impact of US policy on contraceptive access: a policy analysis. Reprod Health.

[6] Grindlay K, Grossman D (2016). Prescription birth control access among U.S. women at risk of unintended pregnancy. J Womens Health (Larchmt).

[7] Mercier AM, Carter SR, Manning N (2022). Racial and ethnic disparities in access to gynecologic care. Curr Opin Anaesthesiol.

[8] NA (2019). Over-the-counter access to hormonal contraception: ACOG committee opinion summary, number 788. Obstet Gynecol.

[9] Landau SC, Tapias MP, McGhee BT (2006). Birth control within reach: a national survey on women's attitudes toward and interest in pharmacy access to hormonal contraception. Contraception.

[10] Greene D, Sharon F, Landau C, Monastersky N, Chung F, Kim N, Melton M, McGhee BT, Stewart F (2006). Pharmacy access to emergency contraception in California. Perspect Sexual Reprod Health.

[11] Lloyd K, Gale E (2005). Provision of emergency hormonal contraception through community pharmacies in a rural area. J Fam Plann Reprod Health Care.

[12] Doshi JS, French RS, Evans HER, Wilkinson CL (2008). Feasibility of a self-completed history questionnaire in women requesting repeat combined hormonal contraception. J Fam Plann Reprod Health Care.

[13] Grossman D, Fernandez L, Hopkins K, Amastae J, Garcia SG, Potter JE (2008). Accuracy of self-screening for contraindications to combined oral contraceptive use. Obstet Gynecol.

[14] Dabrera G, Pinson D, Whiteman S (2011). Chlamydia screening by community pharmacists: a qualitative study. J Fam Plann Reprod Health Care.

[15] Rafie S, Kelly S, Gray EK, Wong M, Gibbs S, Harper CC (2016). Provider opinions regarding expanding access to hormonal contraception in pharmacies. Womens Health Issues.

[16] Harris PA, Taylor R, Minor BL, Elliott V, Fernandez M, O'Neal L, McLeod L, Delacqua G, Delacqua F, Kirby J, Duda SN, REDCap Consortium (2019). The REDCap consortium: building an international community of software platform partners. J Biomed Inform.

[17] Harris PA, Taylor R, Thielke R, Payne J, Gonzalez N, Conde JG (2009). Research electronic data capture (REDCap)--a metadata-driven methodology and workflow process for providing translational research informatics support. J Biomed Inform.

[18] (1970). Minors' Consent to Medical, Dental and Health Services.

[19] (2020). Birth Control. Bedsider.

[20] Tepper NK, Curtis KM, Cox S, Whiteman MK (2020). Update to U.S. medical eligibility criteria for contraceptive use, 2016: updated recommendations for the use of contraception among women at high risk for HIV infection. MMWR Morb Mortal Wkly Rep.

[21] Nielsen J (1994). Estimating the number of subjects needed for a thinking aloud test. Int J Hum Comput Stud.

[22] Sittig DF, Singh H (2010). A new sociotechnical model for studying health information technology in complex adaptive healthcare systems. Qual Saf Health Care.

[23] Strand MA, Bratberg J, Eukel H, Hardy M, Williams C (2020). Community pharmacists' contributions to disease management during the COVID-19 pandemic. Prev Chronic Dis.

